# Neuralgia

**Published:** 1822-12-01

**Authors:** 


1. *Cases of Neuralgia Spasmodica, commonly called Tic Douloureux, successfully treated.* By BENJAMIN HUTCHINSON, Fellow of the Royal College of Surgeons. Second Edition, illustrated with a Plate. Octavo, pp. 189. London, 1822.

2. *A History of a severe Case of Neuralgia, commonly called Tic Douloureux, occupying the Nerves of the Right Thigh, Leg, and Foot, successfully treated.* By G. D. YEATS, M.D. F.R.S. Octavo, pp. 55. London, 1822.

3. *Observations on Neuralgia.* By SHIRLEY PALMER, M.D. New Medical and Physical Journal, Nos. 43 and 53, for May, 1814, and March, 1815.

THE great number of cases of this dreadful disease which have been published of late years, prove that the complaint is on the increase, along with the host of other nervous affections. The general spread of intellectual excitement among all classes of society, in modern times, must deteriorate the grosser functions of the body—and this deterioration inevitably reacts on the nervous system with a severe retaliation. What has been termed neuralgia, or tic douloureux, arises, we are convinced, from different causes in different individuals. It may depend on an inflammatory condition of the neurilema, as has indeed been proved, both by dissection and by the modes of treatment that were successful.* It may depend on organic diseases in the brain, where the origins of the nerves are pressed upon. Or, as is more commonly the case, it may arise from sympathetic irritation of an internal organ, or of a nervous expansion, as that of the inner surface of the stomach or intestines, at a great distance from the seat of the actual pain. It is evident, that the treatment must be modified by this variety in the etiology of the complaint, and that no one specific can ever be expected for the different species of the disease. In that variety of neuralgia originating in gastric or intestinal irritation, tonics have proved the most efficient remedies, combined with, or rather preceded by, evacuations and alteratives. Thus, in two or three of M. Vaidy's cases, (see page 749, vol. i.) cinchona arrested the paroxysms. In other cases, however, (as case 7,) where the patient was young, and the pain in the left leg, along the external cuta-

* See M. Vaidy's cases of Neuralgia, vol. i. p. 748, of this series.

neous nerve, most excruciating, thirty leeches applied in the track of the nerve, removed the disease at once. In the very remarkable case of tic douloureux of the ankle, related at page 354 of the second volume of our *Quarterly Series*, for January, 1820, alterative aperients, and, subsequently, the oxyde of bismuth, taken for a considerable time, effected a cure, or, at least, rendered the complaint of trifling inconvenience. We think the rationale of the tonic plan, where the disease depends on morbid irritability and irritation in the intestinal canal, is not very difficult to understand,—at least, where the mind is not imbued with the modern mania of medical scepticism. We have now, indeed, a very plentiful growth of medical philosophers, whose doubts and difficulties respecting the most common phenomena, and the most obvious trains of causes and effects, must render them, at the bed-side of sickness, as perplexed as the ass between two bundles of hay! We have had a few opportunities of coming in contact with some of these *exquisites* of the profession, who are prone to "cut blocks with a razor," or mystify every thing relating to pathology and practice; and, we must say, that their scepticism and hair-breadth discriminations led them into the most puerile, absurd, or vacillating practice, (if practice it could be called,) which we ever beheld. That great uncertainty does, and ever will prevail in medicine, must be admitted and deplored; but if the absence of *demonstrative proof* in every thing before us, leads us to a want of promptitude in acting on what is *highly probable*, then the practice of medicine will become a tissue of imbecility. We shall, indeed, become "too fond of the right to pursue the expedient," and allow quacks and ignorant pretenders to seize the obvious indications before their eyes, while we are doubting, disputing, and addling our brains with medical logic that, after all, leads but to conclusions in which nothing is concluded. The good practitioner should observe accurately, deliberate maturely, but act decisively on the most *probable* indication before him, without hesitating, or embarrassing himself with the thousand *possibilities* of the case, that may or may not occur.

We shall now take a short review of the history and treatment of this terrible disease, availing ourselves freely of an excellent paper drawn up by our former colleague, (Dr. Palmer,) but unfortunately consigned to a journal which had no circulation, and where, consequently, it has remained unknown. We shall here rescue it from unmerited oblivion.

This obscure and distressing disease has received various appellations, but none, we think, so appropriate as NEURALGIA, first applied in France, and first introduced into this country by Dr. Palmer. It is that which is now generally used by the best writers. It shews the necessity which exists, and ever will exist, for new and more appropriate designations, as we advance in knowledge. By Fothergill, who first described the disease, it was termed, "Faciei morbus nervorum excrucians"—by Sauvages, "tic douloureux"—by Darwin, "hemicrania idiopathica." Although it more frequently affects the three grand divisions of the fifth, and facial portion of the seventh pair of nerves, yet multiplied observations have now proved, that it may assail various other nerves, cerebral, spinal, and, perhaps, ganglionic. Where it is confined to the fifth or seventh pair, *neuralgia facialis* appears a very proper designation.

The scalpel, from utter inefficacy of other local and constitutional treatment, has been often resorted to; yet, this constitutes, at best, but an unpleasant and precarious operation—at one time rendered abortive from the number or situation of the affected nerves—unavailing at another, from defect of anatomical knowledge*—and always disfiguring, more or less, the person operated on.

Where *pain* is a prominent feature in any complaint, we naturally look to the class of anodyne medicines for relief; accordingly OPIUM has been largely exhibited in neuralgia, but with little permanently beneficial influence. In fact, by constipating the bowels, and otherwise deranging the digestive functions, it would appear to be ultimately injurious, rather than sanative. This position is well exemplified in the unfortunate fourth history of neuralgia, related by Dr. Pearson,† where tincture of opium, to the amount of 600 drops, in seven hours, scarcely procured a transient relief. In the cases related by Drs. Darwin,‡ Palmer,§ and Mr. M'Kecknie,|| opium had no salutary effect. Mercury has sometimes succeeded, but oftener failed, in this deplorable disease. In Dr. Pearson's first case, (Ed. Journal for July, 1807,) the disease eventually yielded to the mercurial influence, but not until after a singularly obstinate resistance—the recovery, after all, being slow and imperfect. In the histories recorded by Dr. Darwin and Mr. M'Kecknie, no

---

* If proof of this assertion were wanting, we need only refer to a case of neuralgia recorded by Dr. Darwin, in which the surgeon "*made an incision so as to divide the artery near the centre of the ear, next to the cheek, hoping to divide a branch of the affected nerve, but without success.*" What nerve could he hope to cut by an incision thus directed?

† Ed. Journal, Vol. iii. p. 275. ‡ Zoonomia, Vol. iii. p. 217.

§ New Med. and Phys. Journal, Vol. iii. p. 380.

|| Ed. Journal, Vol. vii. p. 300.

apparent good resulted from the mercury. But where a *combination* of mercury and opium was employed, the prospect is somewhat brighter. Dr. Pearson's second case, and those detailed by Drs. Corkindale and Palmer, present illustrations of the beneficial effects of this combination. It is curious, but it accords with our own observations, that the combination of opium and submuriate taken internally, is far preferable to mercury rubbed on the skin, and opium taken by the mouth. This was conspicuous in Dr. Pearson's and the other cases.

*Arsenic.* From the striking features of analogy observed to subsist between neuralgia and certain forms of chronic rheumatism—and, also, from the periodicity of its attacks, arsenic has been frequently employed, but not with much success. In Dr. Pearson's fourth case, its exhibition proved injurious; in the fifth, unavailing; in Dr. Darwin's patient, useless. In the case detailed by Mr. M'Kecknie, arsenic was prescribed with great and permanent benefit. It should be borne in mind, however, that mercury had been *previously* administered to the point of salivation. Mr. Hill also speaks favourably of arsenic in neuralgic affections.* but cites no case in support of the assertion.

*Cinchona* has been tried in several cases. Dr. Clark, of Nottingham, prescribed it largely, and it suspended the paroxysms for some months, but they eventually returned, with greater violence than ever. In Dr. Darwin's patient this remedy completely failed. Of Mr. Swan's observations on this remedy, and on tic douloureux in general, we have given an ample account in the second volume of this series, p. 63, *et seq.* to which we refer also for Dr. Kerrison's sentiments on the same remedy and disease. They both speak highly of the cinchona.

Dr. Palmer exhibited the bark with much advantage when the system had been worn down by protracted suffering, confinement, and a long course of mercury. It is under such circumstances, he thinks, the cinchona is principally indicated.

*Belladonna.* In the year 1818, Mr. Bailey, of Harwich, drew the attention of practitioners to the use of belladonna in neuralgic affections, and relates nearly thirty cases wherein the medicine was more or less successful. He recommends the extract and tincture of the herb, as prepared by Mr. Corbyn, and details many cases of neuralgia facialis,

* Ed. Journal, vol. vi. p. 57.

where it was more or less beneficial. Of the tincture, he exhibits from twenty to thirty minims for a dose, in any convenient vehicle, augmenting or diminishing it according to its effects, and repeating it with the frequency required by the degree of uneasiness. Of the extract, he begins with three grains, and repeats the medicine in diminished doses, until relief be procured.

Mr. Bailey considers tic douloureux as a *local* disease, having "its origin in the diseased state of the membranes, lining the cavities of the molar teeth." Some others of the profession have reported favourably of this medicine; but, we fear, that it has either not been sufficiently tried, or not been found to answer the expectations of Mr. Bailey.

Among the internal remedies which have been employed against this severe affliction, we should not pass over the plan of Abernethy—alteratives and low diet. This has, unquestionably, in several instances, given great relief, and in a few, we believe, effected a cure; but, still, it was far from being even generally successful. Before coming to the latest remedy—the remedy of the day (carbonate of iron) we may just glance at the principal *external* applications which have been tried in mitigation or removal of this painful malady.

*Leeches*, especially on the Continent, have been the most successful topical application; and where the disease depends on an inflammatory affection of the nerve or its neurilema, as we believe it often does, we can have no difficulty in accounting for the success of local bleeding. Where the disease, on the other hand, has for its cause a constitutional derangement (as no doubt it ofter has) or a morbid sensibility of the nervous system generally, then local bleeding can do little good—nay, it may do positive harm, as appeared to happen in Dr. Yeats's case to be presently noticed. The same observations apply to blisters and other exutories. In the gazette de Santé, for September 1816, Dr. Barras relates a case of neuralgia of the spermatic cord cured by moxa. In this case a periodic but violent hemicrania had been cured five years previously by a blister to the nucha. The same patient afterwards became affected with teazing and lancinating pains in the left epididymis and spermatic cord, inducing, at length, considerable testicular inflammation, which was subdued by the usual means. Thenceforth the pain was uninterrupted, but variable in severity. In the worst paroxysms, the pain radiated to the nates, the left thigh and leg, in the course of the vas deferens, and to the bladder and urethra, inducing frequent desire to void urine, with a scalding in passing it. Leeches, poultices, anodynes, and a caustic issue, gave no relief. Under these circumstances, moxa was applied on the pained part, which partly removed the pain. Another application was made fifteen days afterwards, which effected a cure.

In Mr. Beddingfield's "Compendium of Medical Practice," published in the year 1816, there is related a case of tic douloureux, cured by the application of cerussa, so as to paralyze the nerve affected. This was under the direction of Sir Astley Cooper, in a case which had resisted every other remedy, including the knife. Two scruples of the carbonate of lead were formed into an ointment, and rubbed in the morning on the affected cheek, about an hour before the paroxysm was expected. This application was continued for a month or more, and the man left the hospital cured. The effect of the lead is represented as rapid and striking, the patient being rendered comparatively comfortable in a short time, from a state of excruciating torment. The lead produced no particular effect on the bowels or general system.

*Carbonate of Iron.* About two years ago, Mr. Hutchinson, of Southwell, published a small pamphlet containing six cases of tic douloureux, cured or relieved by carbonate of iron, taken in large doses. Of these cases we gave an account in the first volume of the present series, p. 111, *et seq.* A second edition of this work has just appeared, containing a considerable number of communications from medical practitioners in various parts of the kingdom, bearing testimony, more or less, in favour of the remedy under review. We were a little disappointed, to find that Mr. Hutchinson has published only three or four *additional cases of his own*, in this second edition, (and some of them not of the most satisfactory kind,) though he states, that more than two hundred cases have come under his care since the first edition of his work. We do not think the excuse which he offers is a good one—namely, the fear of swelling out his book. We imagine that the pages of a medical work, could scarcely be better employed, than in recording authentic cases of tic douloureux, especially where a new remedy, of such pretensions as the carbonate of iron, was on trial. We do think that, it is incumbent on Mr. Hutchinson to give these cases, or an abstract of them, to the public, through some medium or other; and to state the unsuccessful, as well as the successful issues of all. We shall now proceed to lay before our readers brief abstracts of the additional facts contained in the volume under review.

*Case* I. This case was tr insmitted to Mr. Hutchinson by an eminent physician in London. A Baronet experienced a first attack of tic douloureux four years ago, and has since suffered four or five paroxysms daily, particularly when washing his face, or eating. The patient was dyspeptic, leucophlegmatic, and of languid circulation. He had tried a variety of remedies. From Mr. Abernethy's plan he had derived, at one time, essential benefit. "When at the sea-side, he has been entirely free from the complaint." Being in great pain from the disease, he began the carbonate of iron, as recommended by Mr. Hutchinson, and felt relief in two or three days. In ten days, he was not only free from pain, but greatly improved in health. He has lately experienced some very slight at tacks; and when the premonitory symptoms occur, he has recourse to the same remedy, which speedily removes them.

*Case* 2. (Communicated by Mr. Richmond, of Grimsby, in Lincolnshire.) Mrs. Vicars, a delicate woman, 30 years of age, suffered for four years with "severe and and acute pain along that branch of the fifth pair of nerves, passing to the maxilla inferior, and particularly affecting the mental nerve." This pain was periodical. She had received an injury on that side of the jaw eleven years before. Various means had been used with little success. A drachm of the carbonate of iron was ordered to be taken three times a day, and on finishing twenty-four powders, she expressed very great amendment," but the iron had produced salivation, (an effect which it frequently has, according to Mr. Hutchinson's experience.) She persevered, however, and a perfect restoration of health was the consequence. Mr. Richmond, in a eulogy on his revered tutor, Mr. Abernethy, expresses his conviction, that this was a local disease from disordered digestion.

*Case* 3. Dr. Carter, physician to the Kent and Canterbury Hospital, has communicated the following case to our author. A man, 58 years of age, who had led an intemperate life, was seized in the middle of October, 1820, with a violent pain, commencing at the upper jaw, and extending, in a short time, over the whole of the left side of the face to the temple. A molaris removed, but no relief followed. Two more teeth were extracted, but the pain became severer than ever. No means gave permanent, and opium only transient, relief. When he presented himself at the hospital, he was in a state of general debility, pulse feeble, tongue foul, bowels constipated. Decoction of bark, with ammoniated tincture of guaiacum every three hours—Dover's powder at bed-time. Three weeks perseverance in this plan produced evident benefit to the general health, but no mitigation of the neuralgia. The carbonate of iron (a scruple every three hours, made into an electuary) was prescribed. In ten days the patient was free from pain, his appearance much improved, and only a numbness of the side of the face remaining. He was ordered to take decoction of bark and aromatic confection to secure against relapse. He continues perfectly well.

*Case* 4. This, and the succeeding case fell under the inspection of Mr. Hutchinson himself.—Mr. Todd, a farmer of Faunfield, 26 years of age, began to have uneasy sensations in the left side of his face about 18 months ago, the pain extending to the ala nasi, upper lip, superior bicuspid teeth, temple, and lip. The infra orbitar nerve and its ramifications appeared to be the seat of the disease, which came on in irregular paroxysms, sometimes mild, sometimes violent, but without any assignable cause. Three molares had been extracted under the idea that the disease originated from them, but without effect. Mr. H. prescribed the carbonate of iron in doses of a drachm three times a day, directing, at the same time, an ointment composed of tartrite of antimony, powdered opium, camphor, and mercurial ointment—one drachm of which was rubbed on the cheek every evening, until a plentiful crop of pustules appeared. It was then suspended, and renewed again when the pustules dried off. This plan was persevered in for three weeks, with very little effect. The medicine now began to produce uneasy sensations in the bowels, which were removed by combining confectio aromatica with the carbonate. During the next six weeks the patient continued gradually gaining ground, "and is now perfectly well."

*Case* 5. Thomas Neep, residing in Southwell, 55 years of age, of scrophulous diathesis and of former intemperate habits, had been subject to tic douloureux during the last ten years. The facial pain was preceded by, and accompanied with giddiness, torpor and other symptoms of determination to the head, which were properly treated by local and general depletion, but without any dimunition of the neuralgia.

"The paroxysms generally began in the upper gums, extending upwards under the eye, diverging towards the ala nasi, and the whole of the right side of the face. The pain was not of the continued, obtuse kind, like that of chronic rheumatism, but, on the contrary, rather transient, exceedingly acute and lancinating during its attack. The periods of its recurrence were indefinite, in the intervals of which he was in tolerable ease. There was some uniformity in the direction and origin of the pain; it always began in the gums and upper lip, and darted upwards towards the orbit: the same sensations were also observed on the bony and fleshy palates, on the gums and teeth of the upper jaw, and sometimes on the fauces." 114.

Nine sound teeth had been extracted at different times, without producing any change for the better; and the same was the result of various internal and external remedies. In the month of March last he came under our author's care, and began to take a drachm of the carbonate of iron twice a day, mixed in honey, and to make use of the emetic tartar ointment, with camphor and powdered opium applied to the face every night. Five weeks careful perseverance in this plan brought some slight alleviation; but the medicine produced considerable diarrhœa, which, however, was checked by a few drops of the tinct. opii. His torments, though mitigated, were still almost insufferable, and therefore the carbonate of iron was increased to four scruples three times a day, in which quantity he persevered for two months, when the violence of the disease began evidently to yield; and after a perseverance in this increased dose for three months, his pains left him. He was seen, "this day, August 28," (this must have been 1821,) when he declared his comforts and his feelings to be "too great for description." Nevertheless the patient, at times, had "slight sensations reminding him of his past sufferings."

Three months after the date of the above report, the patient having been exposed to great vicissitudes and inclemencies of weather, a recurrence of the disease took place, (though not to the same extent as before) and our author could not prevail on him to resume the remedy. He is therefore still suffering at times from his old enemy.

*Case* 6. Mr. Jeffrey Dennis (author of a plan for bettering the condition of seafaring people) writes to Mr. Hutchinson that, having been afflicted with tooth-ache, in the year 1798, he had the tooth broken, and from that time dates the commencement of tic douloureux. Having continued nearly two years, it then disappeared for some years. In 1817, when Mr. Dennis was very busy in planning for the relief of our sailors, his horrible enemy assailed him, and resisted every remedy, till Mr. Hutchinson's pamphlet fell in his way, when he began the carbonate of iron, taking two drachms and two scruples twice a day, from the 26th January, till the 23d July, when he "experienced a complete removal of his agonies." He took it in honey, and made use of opening pills and Epsom salts occasionally to obviate costiveness.

*Case* 7. This was a young lady residing at Mansfield, who had been afflicted with a painful affection of the left side since October, 1818, and which had been gradually increasing in violence for some months. The disease was accompanied with great debility, loss of appetite and sleep, and so great was the dread of the painful side being touched, that she could scarcely bear to be moved in bed. She had consulted the first physicians in England, but with little relief. When she applied to our author, she was suffering the most excruciating pain in her left side, with considerable uneasiness in the right hypochondrium. The pulse was 100, and the debility excessive. "The cutaneous and intercostal nerves appeared to be the principal seat of this truly painful and melancholy affection." The lady commenced the carbonate of iron in the month of November, 1820, and continued it, with some few modifications, until the complaint entirely left her, in the February following. She continued the medicine, however, for some months longer, and up till the present time at intervals.

*Case* 8. Communicated by Mr. Richmond of Grimsby. Mr. Overton, a stout man, turned of 60 years, had suffered severely from repeated attacks of dyspepsia, and general derangement of the digestive organs. About two years ago he began to be affected with an intermitting pain along the inferior maxillary nerve, which gradually increased till it became almost insupportable.

"Mr. Richmond witnessed several paroxysms of pain which produced the greatest agony and distraction, obliging Mr. Overton to run up and down the house with his hands to his mouth, exclaiming violently during the fits, which were very uncertain both in their duration and the succeeding interval, and equally sudden in their accession and departure. In a moment he would exclaim, 'Oh! it's come, or, Oh! there, it's gone:' he then would continue easy for a longer or shorter period. The torment would continue sometimes five minutes, sometime ten, or even longer." 135.

Mr. Richmond gave the patient the carbonate of iron, agreeably to Mr. Hutchinson's plan, "and in ten days he was wholly free from pain, and has now continued exempt from all complaint for more than two months, except that last week he felt a particular cold sensation in his gums, which induced him to send for a repetition of his medicine; there has not, however, been the least return of pain." 136.

*Case* 9. Dr. Alderson, of Hull, communicates a case of neuralgia situated in the female mammæ. The patient was a corpulent woman, 51 years of age, and past menstruation.

Her breasts were so morbidly sensible that the action of passing the hand over them gave exquisite pain, and caused an expression of countenance characterizing tic douloureux. There was no alteration of structure. The carbonate of iron was prescribed, and in a fortnight removed the mammary pain. It returned in a month, and again the medicine (which Dr. A. thinks is as necessary to the patient as her bread,) was resumed. "She has perfectly recovered her former health."

*Case* 10. Is from Dr. Marshall Hall, of Nottingham. The patient was a school-mistress, 58 years of age, who became affected seven years ago with "excruciating pain pursuing the course of the cutaneous nerves, down the outside of the thigh and leg to the sole of the foot." The pain becoming so constant and severe, the patient was obliged to relinquish her school. The operation of dividing the nerves, and all kinds of internal medicine failed. Four months ago, she began to take half a drachm of the carbonate of iron twice a day, which she had persevered in up to the present day—December, 1821. In about six weeks there was some very trifling mitigation of the pain; but it gradually, though almost imperceptibly, yielded to the powers of the iron. The patient's general health improved, her bowels kept regular, and her strength and flesh returned. She has now resumed her scholastic labours.

*Case* 11. This is detailed by the patient himself, Ludovic Houston, Esq. of Johnstone Castle, near Paisley. For five years this gentleman laboured under a nearly constant head-ache, which had been treated as rheumatic, bilious, or nervous, according to the views of the practitioner. We shall give the following sketch of the complaint in the patients own words:

"The headache is confined entirely to the forehead, chiefly seated above the right eye, sometimes the whole of my forehead is affected, and the pain will occasionally remove suddenly to the left temple. I have every day a violent throbbing in both temples, and the pain is sometimes entirely confined to a tooth in the upper jaw, and extending to the nose. The painful sensation is seldom very acute; it continues sometimes the whole day, at others an hour or two, and when the teeth and nose are affected, it seldom lasts more than half an hour, and at times only a few minutes. My digestive organs have all along been very much out of order, and for the last three months, I have taken purgative medicines and mercury to a great extent. There is a peculiarity attending my evacuations, to which I wish very particularly to call your attention. I have voided every day during the above period, immense quantities of slimy stringy mucus, varying from very dark to light colour, and sometimes very black and fœtid. I have generally three evacuations every day, composed entirely of this morbid mucus, and notwithstanding the long continuance of the purgatives, never having omitted them for a day, there is still very little diminution of this excretion." 144-5.

Various remedies were tried, as galvanism, arsenic, cinchona, blisters, local bleeding, and purgatives. The latter class had some good effect. Mr. Hutchinson prescribed a drachm of the carbonate of iron three times a day, accompanied with a tonic mixture of decoct. cinchonæ cum extracto ejusdem. A pill was ordered at bed-time, containing a grain of extr. stramonii, two grains of sulphate of zinc, and three of rhubarb;—the bowels to be kept open by the decoct. aloes;—diet to be nutritious, and some wine to be allowed. From this plan decided benefit was obtained in two months. In the course of six or eight weeks longer the enemy rallied, but with not quite such force as before. The disease has not been eradicated, but the remedy "keeps within very tolerable bounds this foe to his former peace and happiness." 150.

*Case* 12. This who was a patient of Dr. Payne's, (Physician to the General Hospital, near Nottingham) was cured very rapidly of neuralgia faciei, by large doses of the carbonate of iron. He relapsed, and was again relieved from the attacks of the disease by the same remedy.

*Cases* 13, 14. Next follow two cases which happened in the practice of Mr. Cass, a very respectable surgeon of Leeds, communicated by Mr. Hey, of the same town. The first case was that of a female, 54 years of age, who was attacked with tic douloureux on the right side of the face, which resisted the usual plans of treatment. Mr. C. put her on a course of carbonate of iron—half a drachm every four hours, night and day, which was gradually increased till she took a drachm at each dose. No sensible effect resulted during the first fortnight; but afterwards the paroxysms became less violent, and at longer intervals. By a perseverance in the remedy for four or five months the disease was eradicated, and has not since returned.

*Case* 15. Dr. Marsden, of the Nottingham Hospital, has stated a case that occurred in his practice. The patient was a married woman, who had, for some time, laboured under great pain in the course of the left sciatic nerve, which, at length, confined her to her bed. Her strength and flesh were much reduced—pulse, bowels, and catamenia regular, appetite bad. Our author put the woman on a course of carbonate of iron—two scruples thrice a day till the complaint left her, which was in a fortnight or three weeks.

Dr. Ayre, of Hull, has had three well-marked cases of tic douloureux, in which, success was obtained by the carbonate of iron, exhibited in the manner already so often described. A case is also transmitted to our author, by Mr. R. S. Hutchinson, dresser at Guy's Hospital, where a man was cured of tic douloureux by the remedy under consideration. Sir Astley Cooper informs Mr. Hutchinson that, in the few cases in which he had an opportunity of trying the carbonate of iron in tic douloureux, he had observed the benign influence of the medicine over the complaint.

We now come to Dr. Yeats's pamphlet, in which is given a very minute detail of a most painful neuralgic affection, which occurred, we believe, in the person of Mrs. Yeats. This lady awoke, on the morning of March 7th, 1822, with a feeling of uneasiness on the outer ankle, and a little up the calf of the right leg, resembling a cramp, with a numbness of the great toe. During the day, the pain increased, the numbness extending from the toe across the instep. By the evening, the pain had reached the knee, and the whole of that night was passed in much pain. 8th. Fourteen leeches were applied in the line of the pain; but they brought no relief. By the evening, the pain had stretched up the thigh in the line of the sciatic nerve. On this, and also on the preceding night, she had taken four grains of blue pill, followed up the succeeding mornings by a saline aperient. 9th. A blister applied; but the pain still continued to increase, and prove very violent in paroxysms. As the abdominal secretions were evidently deranged, some mercurial medicine, with hyoscyamus and antimonial powder were given every night. On the 12th, the gums were a little affected, but no diminution of the pain was perceptible. The bowels were easily excited, and very sensible;—*the mildest medicine operating and producing pain.* "Their nervous coat had evidently acquired a great morbid sensibility." This, indeed, seemed the case with the sentient extremities of the nerves pretty generally. On the 14th, Sir Henry Halford met Dr. Yeats, and they agreed, that the "disease was a pure affection of the nerves of the leg and thigh, unaccompanied by any morbid state of the muscular and tendinous parts." The pulse was not hurried by any febrile movement, even under the most acute suffering —in general, it was from 56 to 62 in the minute, and soft. It was agreed to keep the bowels open by mild medicine, and lull the pain by narcotics. Little or no relief followed a gentle anodyne diaphoretic;—and the same was the case after considerable doses of hyoscyamus, conium, and pulv. ipecac. comp. Mr. Brodie opened the vena saphena, but very little blood was obtained. The patient, however, thought herself better afterwards. But, still, after this, the returns of pain were frightful. About the 20th March, colchicum was tried; but it irritated the bowels, and produced no good. On the 24th March, the cinchona was commenced, combined with a small quantity of laudanum, and this medicine was continued till the 3d of April, "with decided relief to the pain, and by the acquirement of more comfortable feelings of general health;" but, still, the pain and distress were, at times, so excessive, that it was necessary to give large doses of laudanum, such as 20 drops every six hours. On the 1st and 2d of April she took considerably more than this. The disease having now lasted a month, without any prospect of recovery, it was determined to have recourse to steel, in the manner recommended by Mr. Hutchinson. The subcarbonate, therefore, in half drachm doses, was commenced on the 3d April, and was continued in modified quantities, till the 14th, with considerable mitigation of the pain. At this period, the catamenia recurred, and produced an aggravation, which, however, was but temporary. After this, the medicine was prescribed in the following form, which agreed, far better, with the patient's stomach and bowels:—Pulv. ferri subcarb. ʒss.—p. rad. rhei—p. zingib. ana, gr. ij. ft. pulvis. This was followed by a cordial draught, composed of infus. caryophill. ʒx. sp. ammon. aromat. ʒss. "The happiest effects followed this mode of exhibiting the steel; no more laudanum was taken; the pain began so far to subside, and the paroxysms not to be brought on by pressure, that she slept very well at night; she sat at table at her meals, and was able to resume her domestic functions, with only a reminiscence, by some aching in the limb, that she had had disease there." 18.

" It appears to me," says Dr. Yeats, "to be undoubted, from a review of the case, that the patient is greatly indebted to the Subcarbonas Ferri for her release from this painful complaint, and it is of great importance in the consideration that the sensations of the patient are entirely in its favour. It seemed to have a direct and speedy effect upon the nerves in soothing them by its impression upon the stomach, notwithstanding the large dose in which it was given." 21.

For many interesting observations on neuralgia, and, also, some cases of the disease, which occurred in Dr. Yeats's own practice, we must refer to the work itself.

The length to which this paper has already extended, must render our concluding observations more brief than we originally intended them to be. From all that we have seen and read on the subject, we are inclined to view neuralgia as, in far the greater number of cases, "*a local affection possessing a constitutional origin*;"* or at least intimately connected with a disturbance of the system. Mr. Abernethy states his belief "that this disorder is as much constitutional as either gout or rhematism." The numerous instances now on record, (and, indeed, the facts collected in this article alone,) of the complete and permanent removal of the malady by *internal* remedies, constitute a mass of evidence clearly indicating the constitutional origin of neuralgia. At the same time, we need hardly state that, from this definition, all those forms of the affection, arising from the infliction of external violence, must be obviously excluded. Viewing tic douloureux in this light then, is it, or can it be, ever advisable to divide the nerve? At first sight, it might appear preposterous to have recourse to a surgical operation, if the origin or cause of the disease be constitutional, or, at least, at a distance from the seat of pain. But on a little reflection, it will be remembered that, in organic, as well as inorganized bodies, an effect is frequently seen to become independent, and survive the destruction of its parent cause.† When, therefore, we find the local affection continue unimpaired, when the constitutional disorder, from which it arose, has been removed, we think there would be just grounds for having recourse to division of the nerve. Again, when the local disease becomes unsupportable, from the intensity of the pain, or other attendant phenomena; or, if it appears to react so much on the constitution, as to frustrate the operation of internal remedies, would it not be allowable to obtain by the knife, a truce with the urgent local symptoms, while advantage is taken of the remission to attack the primary disease?

From a review of all the cases hitherto recorded of this painful disease, it appears, that more than three fourths of the patients manifested a deranged state of the digestive organs; and, we conceive that, the salutary operation of all the

---

* Dr. Palmer's Definition. New Med. and Phys. Journal, Vol. IX. p. 177.

† "It will not be overlooked, that it is an object of the highest importance, in all these distressing situations, to endeavour to ascertain any cause that may exist in some distant part, as the fountain of the painful symptoms, and to adopt every feasible method for its removal; but when this is apparently done, complete success is not found uniformly to follow, *for the primary cause of all the original evil may be removed, and still, from new circumstances, habits, &c. the effects remain to keep up extensive pain and mischief.*" Mr. Hill, on Tic Douloureux, Edinburgh Medical and Surgical Journal, vol. vi. p. 57.

most successful remedies, (excepting the narcotics,) consisted in restoring these organs to a better state, and correcting their depraved secretions. We think, however, that the cinchona, and the steel, may owe a great deal of their success in tic douloureux, to their tonic effects on the whole system, by which the morbid sensibility of the nerves, in general, is lessened; and, particularly, the nerves of the chylopoietic apparatus. We apprehend then that, where there is no evidence of local injury, the most rational and successful mode of treatment will be found to consist in evacuations from the stomach and bowels—alteratives—tonics—and anodynes. Of the class of tonics, we think the carbonate of iron seems, from the experience yet acquired, to be the most powerful and successful in tic douloureux, and, therefore, we have no hesitation in saying, that the profession, and society at large, are under deep obligations to Mr. Hutchinson for its introduction as a remedial agent in so direful a malady.

